# *De novo* assembling a high-quality genome sequence of Amur grape (*Vitis amurensis* Rupr*.*) gives insight into *Vitis* divergence and sex determination

**DOI:** 10.1093/hr/uhae117

**Published:** 2024-04-26

**Authors:** Pengfei Wang, Fanbo Meng, Yiming Yang, Tingting Ding, Huiping Liu, Fengxia Wang, Ao Li, Qingtian Zhang, Ke Li, Shutian Fan, Bo Li, Zhiyao Ma, Tianhao Zhang, Yongfeng Zhou, Hongjun Zhao, Xiyin Wang

**Affiliations:** Shandong Academy of Grape, Jinan 250100, China; State Key Laboratory of Southwestern Chinese Medicine Resources, Chengdu University of Traditional Chinese Medicine, Chengdu 611137, China; Institute of Special Animal and Plant Sciences of CAAS, Changchun 130000, China; Shandong Academy of Grape, Jinan 250100, China; Shandong Academy of Grape, Jinan 250100, China; Shandong Academy of Grape, Jinan 250100, China; Shandong Academy of Grape, Jinan 250100, China; Shandong Academy of Grape, Jinan 250100, China; Shandong Academy of Grape, Jinan 250100, China; Institute of Special Animal and Plant Sciences of CAAS, Changchun 130000, China; Shandong Academy of Grape, Jinan 250100, China; Shenzhen Branch, Guangdong Laboratory of Lingnan Modern Agriculture, Genome Analysis Laboratory of the Ministry of Agriculture and Rural Affairs, Agricultural Genomics Institute at Shenzhen, Chinese Academy of Agricultural Sciences, Shenzhen, 518000, China; Shenzhen Branch, Guangdong Laboratory of Lingnan Modern Agriculture, Genome Analysis Laboratory of the Ministry of Agriculture and Rural Affairs, Agricultural Genomics Institute at Shenzhen, Chinese Academy of Agricultural Sciences, Shenzhen, 518000, China; Shenzhen Branch, Guangdong Laboratory of Lingnan Modern Agriculture, Genome Analysis Laboratory of the Ministry of Agriculture and Rural Affairs, Agricultural Genomics Institute at Shenzhen, Chinese Academy of Agricultural Sciences, Shenzhen, 518000, China; Shandong Academy of Grape, Jinan 250100, China; North China University of Science and Technology, Tangshan 063000, China

## Abstract

To date, there has been no high-quality sequence for genomes of the East Asian grape species, hindering biological and breeding efforts to improve grape cultivars. This study presents ~522 Mb of the *Vitis amurensis* (*Va*) genome sequence containing 27 635 coding genes. Phylogenetic analysis indicated that *Vitis riparia* (*Vr*) may have first split from the other two species, *Va* and *Vitis vinifera* (*Vv*). Divergent numbers of duplicated genes reserved among grapes suggests that the core eudicot-common hexaploidy (ECH) and the subsequent genome instability still play a non-negligible role in species divergence and biological innovation. Prominent accumulation of sequence variants might have improved cold resistance in *Va*, resulting in a more robust network of regulatory cold resistance genes, explaining why it is extremely cold-tolerant compared with *Vv* and *Vr*. In contrast, *Va* has preserved many fewer nucleotide binding site (NBS) disease resistance genes than the other grapes. Notably, multi-omics analysis identified one *trans*-cinnamate 4-monooxygenase gene positively correlated to the resveratrol accumulated during *Va* berry development. A selective sweep analysis revealed a hypothetical *Va* sex-determination region (SDR). Besides, a PPR-containing protein-coding gene in the hypothetical SDR may be related to sex determination in *Va*. The content and arrangement order of genes in the putative SDR of female *Va* were similar to those of female *Vv*. However, the putative SDR of female *Va* has lost one flavin-containing monooxygenase (FMO) gene and contains one extra protein-coding gene uncharacterized so far. These findings will improve the understanding of *Vitis* biology and contribute to the improvement of grape breeding.

## Introduction

The grape genus (*Vitis*) contain about 60 species of vining plants. Eurasian grape, East Asian grape, American grape, and other intergeneric hybrid grapes are among the most common grape varieties globally [1].

**Figure 1:**
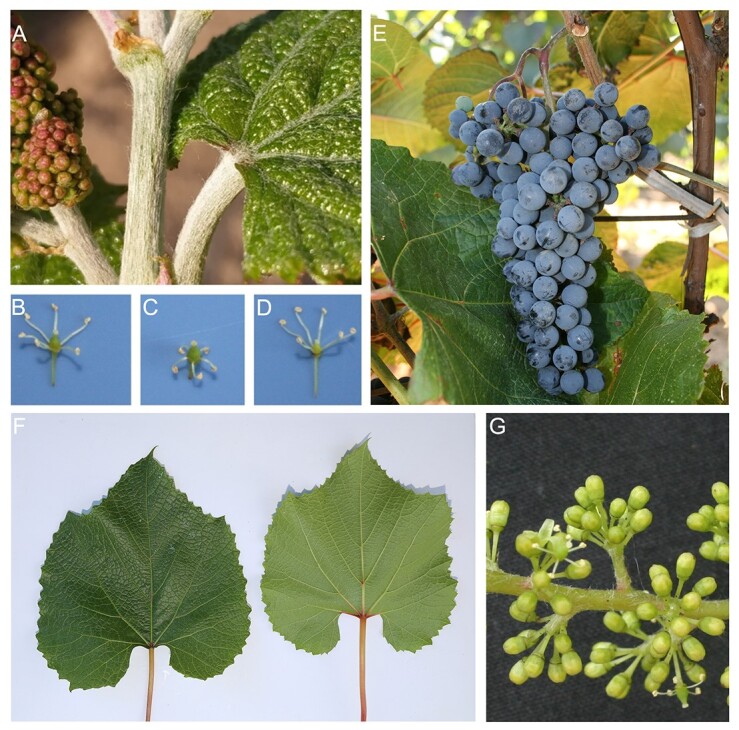
Amur grape. **A** Spider silk-like hairs of Amur grape. **B** Bisexual flower. **C** Female flower. **D** Male flower. **E** Amur grape cv. ‘Zuoshan 1’. **F** Leaf of ‘Zuoshan 1’. **G** Flower of ‘Zuoshan 1’.

As a wild plant, originated in eastern Asia, Amur grape (*Vitis amurensis*, *Va*), also called ‘Chinese wild grape’ or ‘Shanputao’ [2, 3], is one of the East Asian grape species, resembling the wine grape (one of the Eurasian grapes). Wild Amur grapes are found in the Yellow River basin and Songhua River basin in China, Siberia, Russia, Korea, Japan, and many other areas around the world [4]. Amur grapes have female, male, or bisexual plants (Fig. 1B–D). Bisexual grapes are more popular than females because they do not require planting of male plants for pollination. Therefore, the gender of grape flowers is an important topic for breeders and in the grape industry. The Latin name ‘*Vitis amurensis*’ of the Amur grape was first proposed in 1857 by botanist Franz Josef Ruprecht, and the cultivation of *Va* can be traced back to 1907 (wine-world.com). A female cultivated variant of Amur grape called ‘Zuoshan 1’ is widely planted in Northern China. A bisexual Amur grape cultivar, ‘Shuangyou’, is also widely cultivated.

Amur grape has huge commercial potential because of the abundant phenolic compounds, procyanidins, oligostilbenes, and stilbenes it contains [4]. Its fruits are dark purple with small berries, more acidic than those of *Vitis vinifera* (*Vv*), and are used as raw materials for claret, sweet red wine, semi-dry wine, semisweet red wine, and brandy (wine-world.com). Young twigs of the Amur grape have spider silk-like hairs (Fig. 1A). Amur grape is extremely cold-tolerant and can survive temperature as low as −40°C [5, 6]. Amur grape has now become a valuable germplasm resource for grape breeding and wine production. Cultivar ‘Beibinghong’, a cross of *Va* and *Vv*, is a good example of cold resistance, having the ability to tolerate temperatures of −37°C, and is therefore used for preparing fancy red ice wine. Recent pharmacological studies showed that Amur grape possesses anti-inflammatory, antimicrobial, antioxidant, and anticancer properties [4].

The genome sequence of *Vv* cv. ‘Pinot Noir (PN40024)’, a kind of Eurasian grape species, has been deciphered [7–9] and has been used as the reference for genetic and breeding analyses [10, 11]. A high-quality genome sequence of *Vv* cv. ‘Cabernet Sauvignon’ was sequenced in 2021 with Hi-C technology. The genome of *Vitis riparia* (*Vr*), an American grape species, was recently decoded [12]. The *Va* IBCAS1988 genome was sequenced in 2021, and an an assembly of 604.56 Mb and N50 282 256 bp was acquired [3]. To date, there is no high-quality sequence for genomes of the East Asian grape species, hindering biological and breeding research efforts to improve grape cultivars. Here, we present a high-quality genome sequence for the *Va* cv. ‘Zuoshan 1’, which is a well-known Amur grape variety for winemaking in the industry, with its gender and key features well characterized. The present effort will lay a solid foundation for understanding grape biology and developing new breeding lines by deepening our insight into grapes’ evolution and genetic variability, the molecular basis for cold adaptation and sex determination of grapes, etc.

## Results

### A high-quality Amur grape reference genome

The study used the female Amur grape ‘Zuoshan 1’ (Fig. 1E–G) as the material. Its genome sequence was assembled *de novo* with 52.69 Gb (~101.46-fold coverage) of Nanopore reads, 98.79 Gb (~189.23-fold coverage) of MGISEQ short reads, and 58.12 Gb (~111.34-fold coverage) of Hi-C data. A *k*-mer analysis of Illumina reads estimated the Amur grape genome to be ~532 Mb, with DNA heterozygosity 1.20% (Table 1). The final assembled genome sequence reached ~522 Mb, covering ~97.5% of the estimated genome (BUSCO value = 97.5), with a GC content of 0.345 (Table 1).

**Table 1:** Statistics for the *Va* ‘Zuoshan 1’ genome assembly in comparison with the *Va* IBCAS1988 genome.

**Genomic feature**	**Zuoshan 1**	**IBCAS1988**
Estimated genome size (Mb)	532.35	607
Total assembly size (Mb)	522.28	604.56
Number of contigs	615	
Largest contig (bp)	14 947 757	2 623 866
Contig N50 length (bp)	2 512 611	282 256
Scaffold N50 (bp)	26 519 999	748 673
Sequences anchored to chromosomes (Mb/%)	513.47/98.31	
GC content (%)	34.50	34.37
Complete BUSCOs (%)	97.50	
Repeats (%)	59.21	47.06
Protein-coding genes	27 635	32 885
Heterozygosity ratio (%)	1.20	1.23

**Figure 2:**
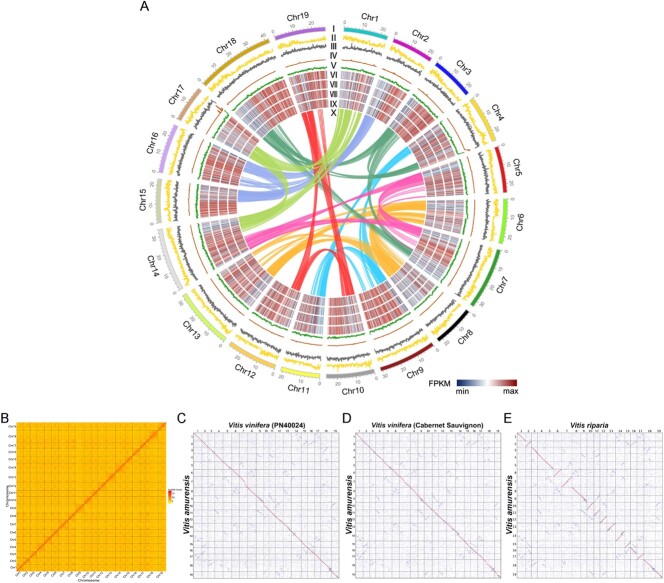
Genomic features of *Va* ‘Zuoshan 1’ and comparison with other genomes. **A** Landscape of genome assembly and annotation of *Va* ‘Zuoshan 1’. Tracks from outer to inner: I, chromosomes; II, gene density; III, repeat density; IV, non-coding RNA density; V, GC content; VI–IX, gene expression levels (FPKM) in S4–S1; X, synteny information. **B** Hi-C map of *Va* ‘Zuoshan 1’. **C** Dotplot of homologous genes between *Va* ‘Zuoshan 1’ and *Vv* PN40024. **D** Dotplot of homologous genes between *Va* ‘Zuoshan 1’ and *Vv* ‘Cabernet Sauvignon’. **E** Dotplot of homologous genes between *Vv* ‘Cabernet Sauvignon’ and *Vr*.

Moreover, the assembly comprised 56 contigs with contig N50 of 2.5 Mb. In comparison, the N50 of *Va* IBCAS1988 is much shorter (282 256 bp) (Wang *et al.,* 2021). The resultant 615 contigs, accounted for 98.31% (~513.47 Mb) of the total assembled genome, anchored onto 19 pseudochromosomes using Hi-C reads (Fig. 2A and B). Repetitive elements make up to 59.21% of the genome sequence (Table 1), much higher than the finding (47.06%) in the *Va* IBCAS1988 genome [3].

We predicted 27 635 protein-coding genes using an integrative strategy combining *de novo* gene prediction, protein-based homology search, and transcript data from RNA sequences of various tissues. Approximately 96.4% of annotated protein-coding genes could be annotated by at least one public database (InterPro, Nr, GO, KOG, Swiss-Prot, TrEMBL and KEGG) (Supplementary Data Table S1). Moreover, 231 miRNAs, 651 tRNAs, 291 rRNAs, and 412 snRNAs were annotated. Long terminal repeats (LTRs) accounted for the largest proportion of transposable elements, making up ~46.39% of the genome.

Homologous gene dot-plotting analysis showed a big difference in the arrangement order of homologous genes between the *Va* ‘Zuoshan 1’ and *Va* IBCAS1988 genomes (Supplementary Data Fig. S1). Moreover, homologous gene dotplots and chromosome synteny analysis showed highly similar arrangement orders of homologous genes across *Va* ‘Zuoshan 1’, *Vv* PN40024, *Vv* ‘Cabernet Sauvignon’, and *Vr* genomes (Fig. 2C–E). *Va* has large inversions on chromosomes 3, 9, 10, and 18 relative to *Vv* and *Vr* (Fig. 2D and E). Besides, *Va* IBCAS1988 chromosomes have large inversions on *Va* ‘Zuoshan 1’ chromosomes 1, 5, 15, 16, 18, and 19 (Supplementary Data Fig. S1). The homologous gene dotplots also showed a major difference between *Va* IBCAS1988 and *Va* ‘Zuoshan 1’ genomes on chromosome 13. The homologous region between chromosome 13 of *Va* IBCAS1988 and *Va* ‘Zuoshan 1’ is clearly divided into two parts. The anterior part of chromosome 13 (~19.6 Mb) of *Va* IBCAS1988 is homologous to the posterior part of the *Va* ‘Zuoshan 1’ chromosome while the posterior part of *Va* IBCAS1988 chromosome is homologous to the anterior part of the *Va* ‘Zuoshan 1’ chromosome (~12.6 Mb) (Supplementary Data Fig. S1). Previous studies on the *Va* IBCAS1988 genome indicated a major difference between *Va* IBCAS1988 and *Vv* PN40024 on chromosome 13 [3]. Here, we found a minimal difference between *Va* ‘Zuoshan 1’ and *Vv* PN40024 on chromosome 13 (Fig. 2C).

### Comparative genomic analysis of three grape species

Duplicated genes produced by the core eudicot-common hexaploidy (ECH) [8] may have contributed to the divergence of the species. We found that *Vr* contains more duplicated genes (5231, 20.04%) than *Va* (4494, 16.26%) and *Vv* (3993, 12.49%) (Supplementary Data Table S2), showing an 800–1300 duplicated gene composition difference. This would have been caused by unbalanced gene losses in the ECH-produced regions. Polyploidization was anticipated to result in genome instability, characterized by extensive DNA rearrangements and gene losses, especially in the early days after the tripling of the genome. Notably, the present finding suggests that the divergent evolutionary patterns among these grapes after the ECH, as much as 130 million years ago (Mya), likely played and still play a non-negligible role in grape species divergence and biological innovation.

**Figure 3:**
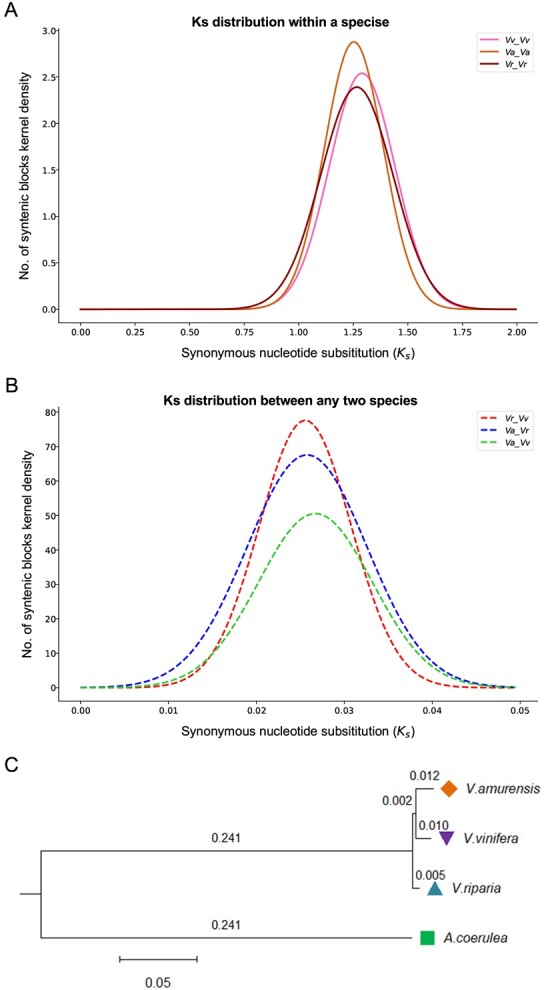
*K*_s_ distribution of collinear genes and phylogenetic relationship of species. **A**  *K*_s_ distribution in each species. **B**  *K*_s_ distribution between any two species. **C** Evolutionary tree of selected plants. The number is the step size and represents the size of the difference between two adjacent sequences.

The synonymous nucleotide substitution rate (*K*_s_) of duplicated genes generated by the ECH shows a difference of up to 3.2% in their evolutionary rates. *Vv*, *Vr*, and *Va* had *K*_s_ peaks located at 1.29 (± 0.150), 1.27 (± 0.165), and 1.25 (± 0.135), respectively, indicating that *Va* evolved the slowest (ANOVA, *P* value = 0.023) (Fig. 3A, Supplementary Data Table 7). The *K*_s_ peaks derived from the orthologous gene pairs between species were located around 0.026 (Fig. 3B, Supplementary Data Table S3). Considering the proposed occurrence time of the ECH, and that the *K*_s_ between the ECH paralogs was ~1.25 (Fig. 3A), we calculated that the three species should have diverged ~2.3–2.8 Mya (the divergence time between *Va* and *Vv* is 2.45–2.77 Mya, that of *Va* and *Vr* 2.37–2.67 Mya, and that of *Vv* and *Vr* 2.35–2.65 Mya) based on the *K*_s_ between orthologs of every two species was ~0.025–0.026 (Fig. 3B).

By using the concatenated multiple sequence alignment of single-copy genes (5929) inferred by OrthoFinder, between the three *Vitis* genomes and *Aquilegia coerulea*, taken as an outgroup, a phylogenetic tree was constructed and showed that *Va* was closer to *Vv* in evolution history (Fig. 3C). When *Arabidopsis* or boxwood was used as an outgroup, we obtained the same inference (Supplementary Data Fig. S2).

**Figure 4:**
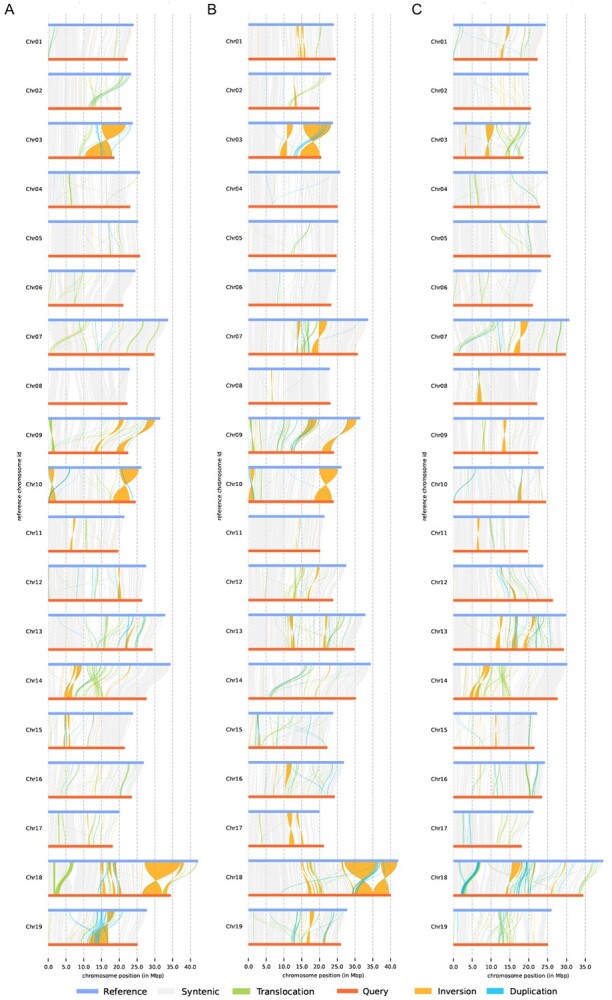
Structural variation analysis. **A** Comparison between *Va* and *Vv*, with *Va* as the reference. **B** Comparison between *Va* and *Vr*, with *Va* as the reference. **C** Comparison between *Vr* and *Vv*, with *Vr* as the reference.

Structural variant (SV) analysis revealed large DNA inversions on *Va* chromosomes 3, 9, 10, and 18 relative to *Vv* (both ‘Cabernet Sauvignon’ and PN40024) and *Vr*, consistent with the homologous gene dotplots (Figs 2 and 4; Supplementary Data Fig. S3). Large DNA rearrangements contributed to the divergence of different grapes. DNA rearrangements in *Vitis* chromosome 19 were inferred across the three species; large inversions were found between *Vr* and *Vv* chromosomes 3, 7, and 18, especially one large DNA segmental translocation detected on *Vr* and *Vv* chromosome 18 (Fig. 4; Supplementary Data Fig. S3).

Genome instability due to the ECH resulted in species-specific regions, also likely to have contributed to their biological divergence. A comparison of *Va* and *Vv* showed that Amur grape had 12 105 specific genomic segments (15.46 Mb), whereas *Vv* had 8465 (11.73 Mb), with 1609 *Va*-specific PAV genes and 79 *Vv*-specific PAV genes positioned within these specific genomic segments (Supplementary Data Table S4). The *Va*-specific PAV genes included those encoding cold-responsive protein kinase 1, ethylene-responsive transcription factor, flavonoid 3′,5′-hydroxylase 2, DELLA2, heat shock 70 kDa protein, methyl-CpG-binding domain protein, and WRKY transcription factor 14 (Supplementary Data Table S5). In contrast, *Vv*-specific PAV genes contained several uncharacterized genes (Supplementary Data Table S6). We found that 16 *Va*-specific PAV genes were cold-resistance-related genes (CRGs). The enrichment of *Va*-specific PAV genes could be related to enhanced biosynthesis of monolayer-surrounded lipid storage bodies (GO:0012511) and stress fibers (GO:0001725) according to GO analysis (*P* < 0.05), and strengthened pathways of calcium signaling (ko04020), and phosphonate and phosphinate metabolism (ko00440) according to KEGG analysis (*P* < 0.05).

A comparison of Amur grape and *Vr* genomes found that *Va* had 7142 genome-specific segments (6.53 Mb) while *Vr* had 3489 segments (7.68 Mb), including 266 *Va*-specific (Supplementary Data Table S7) and 88 *Vr*-specific PAV genes (Supplementary Data Table S8). We found that Vitis03G0393, Vitis11G0466, Vitis16G0074, Vitis14G1585, and Vitis05G0085 from *Va*-specific PAV genes were CRGs. In GO analysis, *Va*-specific PAV genes (*P* < 0.05) were enriched, possibly showing the enhancement of negative regulation of stomatal complex development (GO:2000122), regulation of vacuolar transport (GO:1903335), and cellular water homeostasis (GO:0009992). In KEGG analysis, the enrichment of *Va*-specific PAV genes (*P* < 0.05) may indicate strengthened pathways in biosynthesis of cutin, suberine, and wax (ko00073), and fatty acid (ko00061).

Similarly, we identified 11 503 *Vr*-specific (19.93 Mb) and 5439 *Vv*-specific segments (14.26 Mb), including 1556 *Vr*-specific and 224 *Vv*-specific PAV genes. Actually, a significant difference was found between the numbers of genome-specific segments and the number of specific PAV genes. This indicated prominent differences in DNA and gene retention/loss rates among the three grapes under study.

Amur grape is extremely resistant to coldness. Here, we identified 349 CRGs in *Va*, 437 in *Vv*, and 404 in *Vr*, accounting for 1.26, 1.37 and 1.01% of the total number of genes in each genome, respectively. An *in silico* interaction network of key CRGs in *Va*, *Vv*, and *Vr* was constructed, as previously described [13]. Then, network robustness was calculated after the removal of certain numbers (5 or 10) or percentages (5, 10, or 20) of nodes. Though *Va* had the fewest CRGs, research into the robustness of CRG regulatory networks showed that the CRG regulatory network was more robust in *Va* than in *Vv* and *Vr* (Table 2). This finding may explain why the Amur grape is extremely cold-resistant.

In addition to cold resistance genes, we studied disease resistance genes. We identified totally 64 nucleotide binding site (NBS) genes in *Va*, which is smaller than the number in *Vv* (158 NBS family genes) and in *Vr* (172 NBS genes). Inference of collinear genes showed that 34 *Va* NBS genes (53.13% of *Va* NBS genes) were related to the ECH and 42 (65.63% of *Va* NBS genes) to the tandem duplication. The *K*_s_ value of four pairs of *Va* NBS genes was 0.025 (Supplementary Data Fig. S4), showing the NBS duplicates were likely generated after divergence of *Va* from the other grape species. By inferring gene collinearity across three grapes and *A. coerulea*, we inferred 151 ancestral *Vitis* NBS genes and found that 124 of them might have been lost in *Va*. In contrast, *Vv* and *Vr* were inferred to have lost many fewer NBS genes (57 in *Vv* and 54 in *Vr*). The evolutionary tree of NBS genes supports this inference; it shows that many branches have multiple *Vv* or *Vr* genes but only one or no *Va* genes (Supplementary Data Fig. S5).

### Regulatory mechanisms of nutrient accumulation at four developmental stages of *Vitis amurensis*

Berries of *Va* are nutrient-rich and contain many phenolic compounds, such as anthocyanins and procyanidins, and stilbenes such as resveratrol [4]. Besides, *Va* berries can be used in winemaking because they are rich in sugars and organic acids.

**Figure 5:**
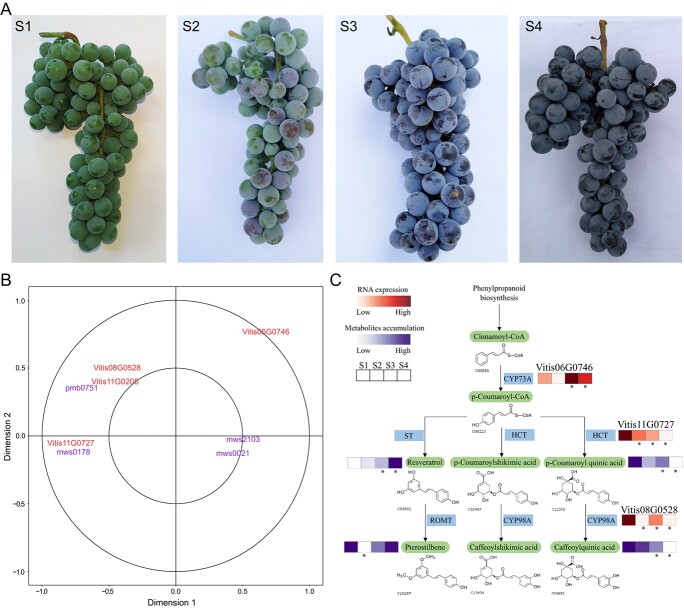
Multi-omics analysis of *Va* fruit at different stages. **A** Fruits at stages S1, S2, S3, and S4. **B** CCA analysis of key genes and metabolites in the resveratrol metabolic pathway. **C** Schematic diagram of resveratrol synthesis and metabolic mechanism during fruit ripening of *Va*. Asterisks indicate that the gene expression or metabolite content of the sample changed significantly compared with S1 (*P* < 0.05). CYP73A, *trans*-cinnamate 4-monooxygenase; ST, stilbene synthase; HTC, shikimate *O*-hydroxycinnamoyltransferase; ROMT, *trans*-resveratrol di-*O*-methyltransferase; CYP98A, 5-*O*-(4-coumaroyl)-d-quinate 3'-monooxygenase.

To reveal the molecular mechanisms of nutrient accumulation in *Va* cv. ‘Zuoshan 1’ berries, we profiled the RNA-seq data (Table 3) and characterized the gene expression in key biological pathways during grape development, and correlations between gene expression and metabolite accumulation. Four developmental stages of berries, including Stage 1 (S1, late period of berry expansion), Stage 2 (S2, veraison), Stage 3 (S3, the period when berries change color completely), and Stage 4 (S4, maturity stage) (Fig. 5A), were analyzed.

The total sugar content increased significantly and titratable acidity content decreased significantly from S1 to S4 (Supplementary Data Fig. S6). Widely targeted metabolomics analysis showed that the contents of succinic acid and 2-isopropyl malic acid decreased significantly from S1 to S4, while the content of d-glucose, a monosaccharide, significantly increased. Trehalose and sucrose, two disaccharides, significantly increased at S3. No significant change was found in tartaric acid content.

To jointly explore gene function with respect to metabolic and transcriptional features, we combined RNA-seq analysis and widely targeted metabolomic analysis. We found that key genes (sucrose-6-phosphatase gene, Vitis08G0202; α,α-trehalase (trehalose synthesis) gene, Vitis02G0204) from the starch and sucrose metabolism pathway (KEGG code Ko00500) were significantly upregulated (>2-fold, *P* value <0.05) at S4 compared with S1. Moreover, we found that at S3 the key genes for sucrose synthesis (sucrose-phosphate synthase genes, Vitis05G0995 and Vitis18G2061) and trehalose synthesis (α,α-trehalase gene, Vitis02G0204) were significantly upregulated. In contrast, the key gene encoding trehalose degradation, β-fructofuranosidase (Vitis16G0541), was significantly downregulated (<0.5-fold , *P* value <0.05) at S3. At S4, the key genes for sucrose synthesis (sucrose-6-phosphatase gene, Vitis08G0202), sucrose-phosphate synthase (Vitis05G0995 and Vitis18G2061), and trehalose synthesis (α,α-trehalase gene, Vitis02G0204) were significantly upregulated. In contrast, sucrose metabolism-related genes (β-fructofuranosidase, Vitis16G0541 and Vitis02G0497) were significantly downregulated at S4 compared with S2.

Widely targeted metabolomics analysis showed that the resveratrol content increased significantly from S1 to S4, but there was no significant change in berries sampled at S1 and S2. As regards the stilbenoid, diarylheptanoid, and gingerol biosynthesis pathway (ko00945), stilbene synthase competitively converted the *p*-coumaroyl-CoA synthesized by *trans*-cinnamate 4-monooxygenase to resveratrol. In contrast, shikimate *O*-hydroxycinnamoyltransferase and 5-*O*-(4-coumaroyl)-d-quinate 3′-monooxygenase could convert the same to caffeoylquinic acid. From S1 to S2, the key genes for caffeoylquinic acid synthesis, the shikimate *O*-hydroxycinnamoyltransferase gene (Vitis11G0727) and the 5-*O*-(4-coumaroyl)-d-quinate 3′-monooxygenase gene (Vitis08G0528) were significantly downregulated. From S1 to S3, the *trans*-cinnamate 4-monooxygenase gene (Vitis06G0746) was significantly upregulated, and the shikimate *O*-hydroxycinnamoyltransferase gene (Vitis11G0727) was significantly downregulated. Meanwhile, the content of caffeoylquinic acid was significantly downregulated, and the resveratrol content increased significantly. From S1 to S4, the *trans*-cinnamate 4-monooxygenase gene (Vitis06G0746) was significantly upregulated, while the shikimate *O*-hydroxycinnamoyltransferase (Vitis11G0727) and 5-*O*-(4-coumaroyl)-d-quinate 3′-monooxygenase (Vitis08G0528) genes were significantly downregulated. Meanwhile, the content of caffeoylquinic acid decreased significantly, while the resveratrol content increased significantly. Canonical correlation analysis (CCA) showed that the *trans*-cinnamate 4-monooxygenase gene (Vitis06G0746), related to the resveratrol content, and the shikimate *O*-hydroxycinnamoyltransferase (Vitis11G0727) and 5-*O*-(4-coumaroyl)-d-quinate 3′-monooxygenase (Vitis08G0528) genes were also related to the caffeoylquinic acid content (Fig. 5B and C).

**Table 2:** Assessment of interaction networks constructed using candidate CRGs in *Va*, *Vr*, and *Vv*.

	All nodes in a network	5	10	5%	10%	20%
*Va*	84	0.851	0.807	0.861	0.826	0.754
*Vr*	91	0.832	0.793	0.839	0.801	0.728
*Vv*	85	0.816	0.782	0.823	0.797	0.731

**Table 3:** Summary of RNA-seq data.

Sample	Clean reads	Mapping rate (%)
S1_1	42 378 612	95.52
S1_2	44 413 474	96.27
S1_3	42 453 694	95.60
S2_1	43 634 464	95.63
S2_2	39 853 380	94.96
S2_3	40 757 404	95.10
S3_1	43 282 574	93.71
S3_2	44 118 914	94.34
S3_3	43 169 656	94.15
S4_1	44 205 082	92.82
S4_2	42 005 090	92.90
S4_3	44 053 722	93.42

Differentially expressed genes (DEGs) were checked (Table 4) between S1 and S4. They included the putative ripening-related gene (Vitis08G0132), the chalcone synthase 2 gene (Vitis05G0886), the flavonoid 3-*O*-glucosyltransferase gene (Vitis16G0156), and the sugar transporter SWEET2a gene (Vitis10G0718), the ethylene-responsive transcription factor 3-like gene (Vitis12G0453), the UDP-glucose:flavonol synthase/flavanone 3-hydroxylase gene (Vitis08G0277), and some bHLH and MYB transcription factors, and WD repeat-containing protein.

### Exploring Amur grape putative sex-determining region

A previous study showed that the *Vv* sex-determining region (SDR) is located on the segment between 4 801 876 and 5 061 548 bp on chromosome 2 and involves 15 protein-encoding genes [14–16]. The 5′ terminus of the SDR segment has a PPR-containing protein-coding gene and the 3′ terminus has an APT3 gene [14]. Another study showed that the SDR is located on the segment between 4 810 929 and 4 921 949 bp on chromosome 2, a subregion of the previously reported SDR [17]. Here, orthologous DNA mapping helped identify a putative SDR in *Va*, between 5 055 465 and 5 198 824 bp on Amur grape chromosome 2. To determine whether the Amur putative SDR is related to sex, we performed the following phylogenetic analysis, population structure analysis, and selective sweep analysis of selected Amur grape plants [18].

**Figure 6:**
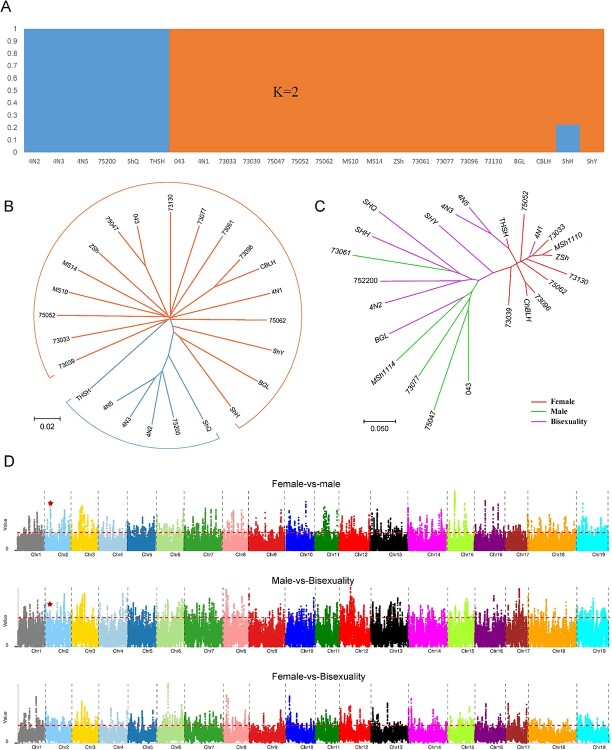
Population structure analysis and selective sweep analysis of 24 *Va* individuals. **A** Population structure of 24 *Va* individuals. Some individuals’ names are abbreviated; refer to Supplementary Data Table S14 for the correspondence between abbreviations and full names. **B** Evolutionary tree reconstructed based on the whole-genome SNPs of 24 *Va* individuals. **C** Evolutionary tree reconstructed based on the SNPs in the SDRs of 24 *Va* individuals **D** Manhattan diagram of the selective region. The top subfigure shows the selective region from the comparison of female and male groups comparison. The middle subfigure is the selective region from the comparison of female and hermaphroditic groups. The bottom subfigure shows the selective region from the comparison of male and hermaphroditic groups. The red dashed line represents the threshold of θπ ratios and FST (top 5%). Red asterisks mark SDRs.

By identifying SNPs in 24 Amur grape individuals, including 5 male, 10 female, and 9 hermaphroditic individuals (including 4 tetraploids), we performed phylogeny analysis and examined genetic population structure among the Amur plants (Supplementary Data Table S9). Using 2 588 125 inferred high-quality SNPs, we explored the relationship among these 24 *Va* individuals and the *Va* reference genome (this study). The phylogenetic tree that we constructed indicated that the individuals could be divided into two groups. However, the grouping proved unrelated to sex differences (Fig. 6A). Then, using the above-inferred SNPs, we performed a genetic population structure analysis and showed that the individuals could be divided into two groups, too. This grouping also proved unrelated to sex division (Fig. 6B). Alternatively, based on the SNPs in only the inferred SDR regions of the 24 individuals, we constructed a phylogenetic tree. Notably, we found that the tree had groups consistent with sex division (Fig. 6C).

Then, a selective sweep analysis was performed according to sex groups. One of the selected regions inferred (both θπ ratios and *F*_ST_ in the top 5% range) from the comparison of male and female accessions totally overlapped the Amur grape putative SDR. We found that the Amur grape putative SDR contains 16 genes (Vitis02G0494, Vitis02G0495, Vitis02G0496, Vitis02G0497, Vitis02G0498, Vitis02G0499, Vitis02G0500, Vitis02G0501, Vitis02G0502, Vitis02G0503, Vitis02G0504, Vitis02G0505, Vitis02G0506, Vitis02G0507, Vitis02G0508, and Vitis02G0509), homologous to *Vv* SDR genes associated with sex (Fig. 6D). Another selected region inferred between male and hermaphroditic groups overlapped part of the Amur grape putative SDR gene Vitis02G0494 (Fig. 6D). However, none of the selected regions inferred between female and hermaphroditic groups overlapped the Amur grape putative SDR (Fig. 6D).

**Table 4:** Number of DEGs.

Group	All	Up	Down
S2 vs S1	3307	1367	1940
S3 vs S1	4633	1862	2771
S4 vs S1	5969	2209	3760
S3 vs S2	2904	1196	1708
S4 vs S2	4817	1904	2913
S4 vs S3	1797	652	1145

Previous studies showed that the SDR of *Vv* M and H haplotype genes include those encoding PPR-containing proteins, a YABBY transcription factor (VvYABBY3), a VviSKU5 (Skewed5), a β-fructofuranosidase, an aldolase, a trehalose-6-phosphate phosphatase (TPP), an inaperturate pollen1 (VviINP1), an exostosin family protein, KASIII, two TPR-containing proteins, a PLATZ transcription factor, three flavin-containing monooxygenases (FMOs), a hypothetical protein (VviFSEX), a WRKY transcription factor, and an adenine phosphoribosyltransferase (VviAPT3). The SDR of *Vv* F haplotypes included genes encoding a PPR-containing protein, a YABBY transcription factor (VviYABBY3), a VviSKU5, a β-fructofuranosidase, an aldolase, a TPP, an inaperturate pollen1 (VviINP1), an exostosin family protein, a KASIII, and a PLATZ transcription factor. The F haplotypes also had four flavin-containing monooxygenases, a hypothetical protein (VviFSEX), a WRKY transcription factor, and an adenine phosphoribosyltransferase (VviAPT3) [14].

**Figure 7:**
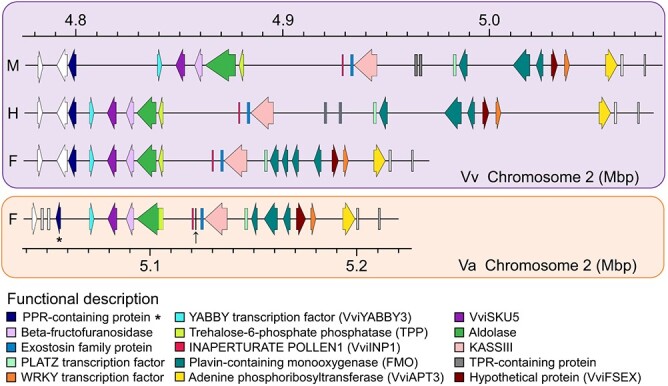
Genes of *Vv* and *Va* SDRs. Genes of *Vv* H, F haplotype SDR are from *Vv* ‘Cabernet Sauvignon’. Genes of *Vv* M haplotype SDR are from *Vv. sylvestris*. Genes of *Va* F haplotype are from ‘Zuoshan 1’. The gene indicated with an arrow is the *Va* uncharacterized protein gene. The key *Va* PPR-containing gene Vitis02G0494 is indicated with an asterisk.

In *Va*, the F haplotypes included 16 genes encoding a PPR-containing protein, a YABBY transcription factor (VviYABBY3), a VviSKU5, a β-fructofuranosidase, a fusion protein of aldolase and TPP, an inaperturate pollen1 (VviINP1), an uncharacterized protein (homologous to VIT_202s0154g00120 of *Vv* PN40024), an exostosin family protein, a KASIII, and a PLATZ transcription factor. Besides, the F haplotypes contained three flavin-containing monooxygenases, a hypothetical protein (VviFSEX), a WRKY transcription factor, and an adenine phosphoribosyltransferase (VviAPT3). The SDR of *Va* F haplotypes had an additional uncharacterized protein gene (Vitis02G0500) and had lost one FMO gene compared with the *Vv* ‘Cabernet Sauvignon’ F haplotypes (Fig. 7).

In *Vv*, the female-specific DNA polymorphisms at −13, −5, and + 2 bp around the transcript initiation sites may reduce transcription and/or alter mRNA decay of the female PLATZ transcription factor allele on the *Vv* SDR [19]. However, *Va* lacks these DNA polymorphisms upstream of the ATG of the PLATZ transcription factor gene in the Amur grape putative SDR. In other words, there is no difference in DNA polymorphisms between female, hermaphrodite, and male *Va* individuals within 20 bp upstream of the PLATZ transcription factor gene.

We found that some other selected regions may be related to sex-determination (θπ ratios and *F*_ST_ estimates in the top of 5%), which include the region (chr3:6 730 001–8 840 000 bp) from the comparison of male and female accessions, the region (chr16:3 565 001–4 830 000 bp) from the comparison of male and female accessions, the region (chr16:15 590 001–18 015 000 bp) from the comparison of male and female accessions, and the region (chr17:11 450 001–13 785 000 bp) from the comparison of male and hermaphroditic accessions. In these regions, there are the MADS-box protein AGL62 genes (Vitis03G0709, Vitis03G0710, Vitis03G0711, and Vitis03G0712) from chr3:6 730 001–8 840 000 bp. Notably, with the exception of the region in chromosome 3, the PPR-containing gene (Vitis02G0494) was found to have one or two homologs in three of the above inferred SDR-related regions, including Vitis16G0214, Vitis16G0215, Vitis16G0545, and Vitis17G0924.

## Discussion

*Vitis* mainly includes the Eurasian grape, the East Asian grape, and the American grape. The sequence of the *Vv* genome (PN 40024), of a Eurasian grape, was published in 2007 [8] and involved research to understand grape biology at the genome scale [2, 10, 20, 21]. Given that its genome has only undergone one polyploidization, the ECH, after splitting from the basal eudicots, it is often used as an outgroup reference for studying the other eudicot genomes [20]. The sequence of the *Vr* RGM genome, of an American grape, was published in 2019 [12]. The genome sequence of ‘Cabernet Sauvignon’, a *Vv* variety, was later published, being assembled using Hi-C technology [14].

Here, we assembled the genome of *Va*. The above analysis showed that the assembled *Va* genome sequence reported here is much improved over the previous one. Based on the present genome sequence, we discovered a likely assembly error in chromosome 13 of the previous grape genome sequence.

The availability of multiple grape genome sequences allows the gene sequences of several grape species to be compared and analyzed so that we could detect the evolutionary history and other important comparative genomics information across the grapes. The work has not been well performed in previous studies. In the present study, gene collinearity analysis showed that the arrangement order of orthologous genes between *Va*, *Vv*, and *Vr* genomes are highly conservative. We have to note here that genome instability due to the ECH, as ancient as 130 million years, contributed to the divergence of the grape genomes. Actually, a characterization of homologous genes showed that *Vr* is the most conservative one among the three. *Vr* contains more paralogous genes produced by the ECH, compared with *Va* and *Vv*, showing the conservativeness of the *Vr* genome, and implying that *Vr* may greatly resemble the common ancestor. Moreover, more orthologous genes were preserved between *Vr* and the other two grapes (*Vv* and *Va*) than between *Vv* and *Va*. Besides, phylogenetic analysis supported the idea that *Vr* was the first one to split from the other grapes. This shows that the *Vr* genome may be taken as a better reference for *Vitis* biology and even that of all the eudicots, in that previously *Vv* was often used to understand the evolution of the other eudicot plants [17]. The present finding that the 130-Mya polyploidization should have contributed to the species divergence of grapes, due to divergent retention/loss of DNA segments and functional genes, reminded us that it may have played a non-negligible role in grape divergence and genetic innovation, similar to what has been found in rice and other grasses [22]. Possibly, the genomic features of thousands of duplicated genes in crops could be manipulated to breed high-yield and/or high-quality crops.

Species-specific genomic segments also make up a large proportion of the genomes of all the three species, implying significant genomic differences among the three species. SV may generate phenotypic differences [23], e.g. *Va* being more cold-resistant than the other two species. Biased sequence preservation in *Va* may have contributed to its ability to resist coldness. Sequence variations, especially incurred by the ECH, may generate phenotypic differences. Here, we found that the *Va*-specific PAV genes contain CRGs, such as those encoding the ethylene-responsive transcription factors and cold-regulated 413 inner membrane proteins. The robustness of the CRG regulatory networks in *Va* is higher than that of those in *Vv* and *Vr*. This shows that the stronger cold resistance of *Va* may be related to the preferential preservation of cold-related genes, and *Va* cold-related genes may constitute a more robust and effective interaction network. A study of CRGs in angiosperms, including *Vv*, supported the idea that their expansion was significantly related to the occurrence of polyploidization [13]. The present study provides further evidence that the ECH, though it occurred 130–150 Mya, still contributes to the enhancement of resistance to coldness in plants.

Multi-omics analysis of the *Va* was performed to understand the mechanism underlying the regulation of nutrient accumulation in the *Va* over its developmental period. *Va* is sourer than *Vv*, a factor that needs urgent resolution by breeders, in that high sourness makes the fruits taste unpleasant and the wine tastes unbalanced. The present analysis revealed that the total sugar increased continuously during ‘Zuoshan 1’ berry development, while the total sugar content in this species/variety was almost equal to that in *Vv*. Further analyses revealed that the total acid content decreased significantly during ‘Zuoshan 1’ berry development. Wide target metabolome analysis showed no decrease in tartaric acid content during ‘Zuoshan 1’ berry development, while contents of citric acid and several malic acid types decreased significantly in ‘Zuoshan 1’. Combined transcriptome and metabolome analyses revealed that sucrose-6-phosphatase, α,α-trehalase, and β-fructofuranosidase genes jointly regulate the accumulation of sucrose and trehalose during ‘Zuoshan 1’ development. The DEGs during S1–S4 comprise the ripening-related protein-like gene, the chalcone synthase gene, which is the first enzyme activated in the flavonoid biosynthetic pathway [24], the UDP-glucose:flavonoid 3-*O*-glucosyltransferase gene, which promotes anthocyanin accumulation in grape [25], the SWEET gene, which is a sugar transporter and uniporter gene [26], the ethylene-responsive transcription factor 3-like gene, containing bHLH, MYB transcription factor genes, and the WD repeat-containing protein genes. Most of these genes may be involved in *Va* berry ripening. Therefore, we infer that these DEGs may play a central role in the process of *Va* berry ripening.

The *Va* genome sequence was further analyzed to understand the relationship between different *Va* phenotypes and genotypes. Given the scarcity of hermaphroditic *Va* in nature, we explored the SDR in this grape. Previous studies have uncovered the SDR of *Vv* [14]. The three *Vv* types (varieties) are determined by the genotype at the SDR. Males are heterozygous for the male and female haplotypes (MF), females are homozygous (FF), and cultivated *Vv* hermaphrodites are either homozygous for hermaphrodite haplotypes (HH) or heterozygous (HF). Previous studies have shown that the SDR of *Vv* M and H haplotypes have two TPR-containing protein genes and three FMOs, and the SDR of *Vv* F haplotypes contains four FMOs without a TPR-containing protein gene [14]. This implies that the type or number of genes in the SDR of grapes varies between sexes.

Further analyses to clarify whether SDR is also present in *Va* revealed that one fragment from *Va* (chr2:5 055 465–5 198 824 bp) is homologous to *Vv* SDR in *Va* (chr2:4.8–5.06 Mbp) and call it the Amur grape putative SDR. In the present study, we sequenced the female *Va* genome. Therefore, further analyses were performed to explore whether the types and numbers of genes in the female *Va* putative SDR were consistent with those in the female *Vv* SDR. Selective sweep analysis of male, female, and hermaphroditic individuals revealed that one selected region from the comparison of male and female accessions overlapped the Amur grape putative SDR including 16 genes all homologous to *Vv* SDR genes (such as the APT3 gene, the PLATZ transcription factor gene, and the TPP gene). A previous study inferred that the VaAPT3 gene (orthologous to Vitis02G0508 in the present *Va* genome), located in the Amur grape putative SDR, was associated with sex determination [27]. Recently, the PLATZ transcription factor gene has been found to determine the grape's sexuality [19]. The TPP gene, also located in the Amur grape putative SDR, and TPP genes in many species have also been associated with sex determination, flower development, reproduction, and synthesis of plant hormones, among other functions [28, 29]. PtTPPs displayed a specific expression pattern in seven developmental stages of *Populus* male and female floral buds [30]. TPP controls inflorescence architecture in maize through sugar signal modification [31]. This suggests that the hypothetical *Va* SDR may be related to sex determination. One selected region from the comparison of male and hermaphroditic accessions overlapped part of the Amur grape putative SDR including only one gene (PPR-containing gene), which is homologous to the *Vv* SDR gene PPR-containing gene. Both the selected region in chromosome 2 from the comparison of male and female accessions and the selected region in chromosome 2 from the comparison of male and hermaphroditic accessions contain the PPR-containing gene Vitis02G0494. This indicated that the gene may be the key gene related to sex determination in *Va*. Additionally, some selected regions may be related to sex determination in *Va*. Previous studies indicated that some PPR-containing proteins genes could restore fertility to cytoplasmic male-sterile plants [32–35]. A study on RNA-seq analysis of three flower sex types in grapevine showed that some grape PPR-containing genes could be essential for carpel development [36]. The study also inferred that a PPR-containing gene(s) may be essential for the perfect development of sexual floral organs. Here, we found that some other selected regions comprise many PPR-containing protein genes, allowing further exploration of their likely contribution to sex determination.

However, no selected region that overlapped the Amur grape putative SDR from the comparison of male and hermaphroditic accessions was found. We found that the type and arrangement order of all genes in the putative SDR of female *Va* and the SDR of *Vv* were similar. However, compared with *Vv* F haplotypes, the putative SDR of female *Va* has lost one FMO gene and contains one extra uncharacterized protein. The uncharacterized protein gene has never been found in the SDR of *Vv* ‘Cabernet Sauvignon’ and *Vitis sylvestris* [14]. The role of the uncharacterized protein gene in *Va* sex determination needs further exploration.

Yang *et al*. studied sex determination based on another East Asian grape, *Vitis pseudoreticulata* ‘Huadong1058’ and found a key SDR between 3.29 and 5.78 bp on chromosome 2 [37]. This indicated that chromosome 2 of many *Vitis* plants may be related to sex determination.

In the other selective region (chr17:11 450 001–13 785 000 bp), we found from comparison of *Va* male and hermaphroditic accessions that there are many MADS-box protein AGL62 genes. Previous studies inferred MADS-box protein genes may be also related to developing male and female flowers [38, 39]. We consider that the region (chr17:11 450 001–13 785 000 bp) may be also related to *Va* sex determination. In addition to those selective regions that have been found to contain PPR-containing proteins gene, it should be explored whether *Va* sex may be determined by several different genomic regions in the future.

## Materials and methods

### Plant materials and DNA sequencing

Fresh leaves and stems of *Va* cv. ‘Zuoshan 1’ were sampled for DNA extraction and sequencing. Total genomic DNA was extracted using the CTAB (cetyltrimethylammonium ammonium bromide) method [40]. The library for ONT sequencing (Oxford Nanopore Technology, Oxford, UK) was constructed using large (>15 kb) DNA fragments with the SQK-LSK109 Ligation Sequencing Kit and sequenced using the ONT platform. Adapters and low-quality nucleotides (with mean quality score <7) were trimmed off. Paired-end libraries with 350-bp insert sizes were constructed following the manufacturer’s protocols and sequenced using the MGIseq 2000 platform (MGI Tech Co. Ltd, Guangdong, China). The MGIseq reads were filtered using the SOAPnuke1.5.6 online software (https://github.com/BGI-flexlab/SOAPnuke). For high-throughput chromosome conformation capture (Hi-C) analysis, fresh leaves and stems of *Va* cv. ‘Zuoshan 1’ were treated following previous methods [41].

### Genome size and heterozygosity estimation

The genome size was estimated from the MGIseq reads using *k*-mer analysis, and the *k*-mer depth-frequency distribution was generated using Jellyfish software [42]. Genome size and heterozygosity were calculated using GenomeScope software [43].

### *De novo* genome assembly

Long ONT reads were corrected using Canu (https://github.com/marbl/canu/releases), and *de novo* assembled using Smartdenovo software (https://github.com/ruanjue/smartdenovo). Racon (https://github.com/isovic/racon) and Medaka (https://github.com/nanoporetech/medaka) software were applied to polish the assembled contigs. The polished, assembled data were then corrected using Pilon (v.1.22, https://github.com/broadinstitute/pilon/). Next, the HaploMerger2 pipeline reduced the assembly to ~522 Mb. After that, the purged assembled contigs were anchored into 19 pseudochromosomes using Juicer with the parameters -g draft -s MboI and the 3D de novo assembly (3D-DNA) pipeline with the parameters -m haploid -r 2 [44, 45].

### Genome annotation and gene prediction

Transposable elements were predicted using combined homology-based comparisons with RepeatMasker v4.0.7, RepeatProteinMask v4.0.7, and *de novo* approaches with Piler [46] (http://www.drive5.com/piler/), RepeatScout, and RepeatModeler. Tandem repeats were identified using Tandem Repeats Finder v4.09 [47] (http://tandem.bu.edu/trf/trf.html) and LTR_FINDER v1.06 [48]. Furthermore, the *Va* protein-coding gene set was deduced by *de novo*, homology, and evidence-based gene prediction (transcriptome data) [49]. The transcript evidence included transcripts assembled from the RNA-seq data of different tissues (leaf, stem, flowers, tendril, and fruit; these samples were mixed and subjected to RNA-seq). Predicted protein-coding genes were finally screened, and sequences of <30 bp or with ≥90% repetitive DNA were filtered out. Next, the predicted genes were functionally annotated using a previous method [49]. Moreover, tRNA was identified using tRNAscan-SE 1.3.1 [50] (http://lowelab.ucsc.edu/tRNAscan-SE/), BLASTN identified rRNA, while INFERNAL (http://infernal.janelia.org/) identified miRNA and snRNA.

### Genome homology inference

Protein sequences from one plant were searched against itself and those of another plant genome using BLASTP [51] to find the best, second best, and other matches with E value <1E−5. Dotplots were produced using the WGDI package of Python [52]. Collinear genes were inferred using the -icl subprogram contained in the WGDI package with default parameters. Nucleotide substitution rates were estimated between collinear homologous genes using the YN00 program in the PAML (v4.9h) package implementing the Nei–Gojobori approach [53].

### Species tree

A rooted species tree was inferred using Orthofinder (version 2.5.4) with the -M msa parameter [54, 55] based on 5929 single-copy genes.

### Identification and characterization of cold-related genes

The cold-related genes in the three grape species and gene network robustness were determined following previous methods [13].

### Structural variation analysis

The SV analysis was performed using the NUCmer program embedded in MUMmer with the parameters -mumreference -g 1000 -c 90 -l 40 [56]. Genomic-specific segment analysis was performed as follows. First, the query genome sequence was split into 500-bp windows with an overlapping step size of 100 bp, and then all the 500-bp window subsequences were aligned against the reference genome by BWA-MEM with the parameters -w 500 -M. The 500-bp windows that failed to align or aligned with <25% coverage were defined as genomic-specific segments. Genes within the genomic-specific segments were defined as the specific genes of the query genome (species-specific PAV genes). SV plots were drawn using ggplot2.

### Widely targeted metabolomic analysis

Berries from four growth stages were used for this study, including Stage1 (S1, the late period of berry expansion), Stage 2 (S2, veraison), Stage 3 (S3, the period when berries change color completely), and Stage 4 (S4, maturity stage). Three biological replicates from each stage were used for metabolome analysis. Metware Biotechnology Co., Ltd (Wuhan, China) performed metabolome extraction and analysis using previously described methods [57]. Metabolites were identified using the Metware database (MWDB), and metabolite abundances were determined according to the metabolite peak areas. Metabolites were considered as differentially accumulated when the variable importance in projection (VIP) was ≥1, and the absolute log_2_ (fold change) was ≥1. Metabolic pathways were constructed according to the KEGG database.

### Total sugar and titratable acidity content analysis

Berries from four growth stages (S1, S2, S3, and S4) were ground into homogenate and used for this study. Three biological replicates from each stage were used for metabolome analysis. We analyzed the total sugar content using the sulfuric acid-anthrone colorimetric method and the instrument used was a Lambda 365 ultraviolet/visible spectrophotometer. We analyzed the titratable acidity content using acid–base titration and using sodium hydroxide standard solution (0.05 mol/l).

### RNA sequencing

Just as we analyzed the metabolome, berries from four stages, S1, S2, S3, and S4, were used for the RNA-seq analysis. Three biological replicates from each berry stage were used for RNA-seq analysis. Novogene Co. Ltd performed the RNA-seq experiments on the Illumina Novaseq 6000 platform (Illumina, CA, USA) following the manufacturer’s instructions and previously described steps [58]. All paired-end reads were mapped to the *Va* genome using Hisat2 v2.0.5. Expression levels were calculated using the FPKM (fragments per kilobase of exon model per million mapped fragments). The DEGSeq R package (1.20.0) was used to identify DEGs. Genes with adjusted *P* value <0.05 and fold change ≥2 or ≤−2 were identified as DEGs.

### Integrated analysis of metabolome and RNA sequencing

For the combined analysis of all metabolome and transcriptome data, CCA was performed on the metabolome and RNA-seq data using the CCA package in the R statistical environment [59]. Next, WGCNA (weighted correlation network analysis) was performed on all RNA-seq and metabolome data using the WGCNA package in the R statistical environment.

### Population genetic analysis

Essentially, 24 Amur grape genotypes were re-sequenced based on the *Va* genome assembled in this study and used for calling SNPs. Concurrently, the ANNOVAR package was employed for population genetic analysis. Clean read data (10-fold *Va* genome size) of 24 kinds of Amur grape were separately obtained on the MGI2000 platform. The SNPs in linkage disequilibrium were filtered using PLINK with a window size of 50 SNPs (advancing 5 SNPs at a time) and a 0.5 *r*^2^ threshold. PCA was conducted using GCTA (v1.25.2) [60]. Furthermore, population structure was analyzed using FRAPPE, and the MLtree was constructed using SNPhylo [61] to clarify the phylogenetic relationships.

### Selective sweep analysis

Selective sweep analysis was performed using a previously described method [62]. In brief, *F*_ST_ and θπ were used to detect candidate selective regions between the three grape populations, *F*_ST_ and θπ were calculated using PopGenome [63, 64] regions with both θπ ratios and *F*_ST_ estimates in the top of 5% were considered as the selection regions.

## Acknowledgements

This research was supported by the Key Research and Development Project of Shandong Province (2022CXGC010605; 2021LZGC025), the Improved Variety Program of Shandong Province (2022LZGCQY019; 2020LZGC008), Shandong Natural Science Funding (ZR2020QC145; ZR2023QC239; ZR2021QC022; ZR2022QC076; ZR2023QC249; ZR2023QC217), the Scientific Research Guide Foundation of Shandong Academy of Grape (SDAG2021A01; SDAG2021A02; SDAG2021B11), the Shandong Academy of Agricultural Sciences, Introduction and Training of High-level Talents (CXGC2022E15), the National Horticulture Germplasm Resources Center-Amur Grapevine Germplasm Resources Branch Center (NHGRC2022-NH04), and the Species and Variety Germplasm Resources Protection Project (2130135).

## Author contributions

P.W., Y.Z., Y.Y., H.Z., and B.L. designed the experiments. T.D., H.L., F.W., S.F., K.L., and Q.Z. performed the experiments and wrote the manuscript. F.M., A.L., Z.M., and T.Z. analyzed the data. J.J., Y.Z., and X.W. edited the manuscript.

## Data availability

The *Va* genome project was deposited at NCBI under BioProject number PRJNA868106 and BioSample SAMN30308461. All *Va* coding sequences, protein sequences, and gff files can be download from the TCMPG 2.0 database under number TCMPG20325 (http://cbcb.cdutcm.edu.cn/TCMPG2/genome/details/?id=TCMPG20325). The *Va* IBCAS1988 genome data can be downloaded from the NGDC database (https://ngdc.cncb.ac.cn/search/?dbId=gwh&q=PRJCA001564&page=1). *Vitis vinifera* cv. ‘Cabernet Sauvignon’ genome data can be downloaded from the grapegenomics.com database (https://www.grapegenomics.com/pages/VvCabSauv/download.php). *Vitis riparia* genome data can be downloaded from the NCBI database (https://www.ncbi.nlm.nih.gov/datasets/genome/?taxon=96939).

## Conflict of interest

The authors declare no competing interests.

## Supplementary data

Supplementary data is available at *Horticulture Research* online.

## Supplementary Material

Web_Material_uhae117
