# Supplementary material for: De novo assembling a high-quality genome sequence of Amur grape (Vitis amurensis Rupr.) gives insight into Vitis divergence and sex determination
Source: Hortic Res. 2024 Apr 26;11(6):uhae117. doi: 10.1093/hr/uhae117 (PMC11197301; doi:10.1093/hr/uhae117)
Supplement: Web_Material_uhae117 [file web_material_uhae117.zip › Supplementary_Figures.docx]

**Supplementary Figures**

**
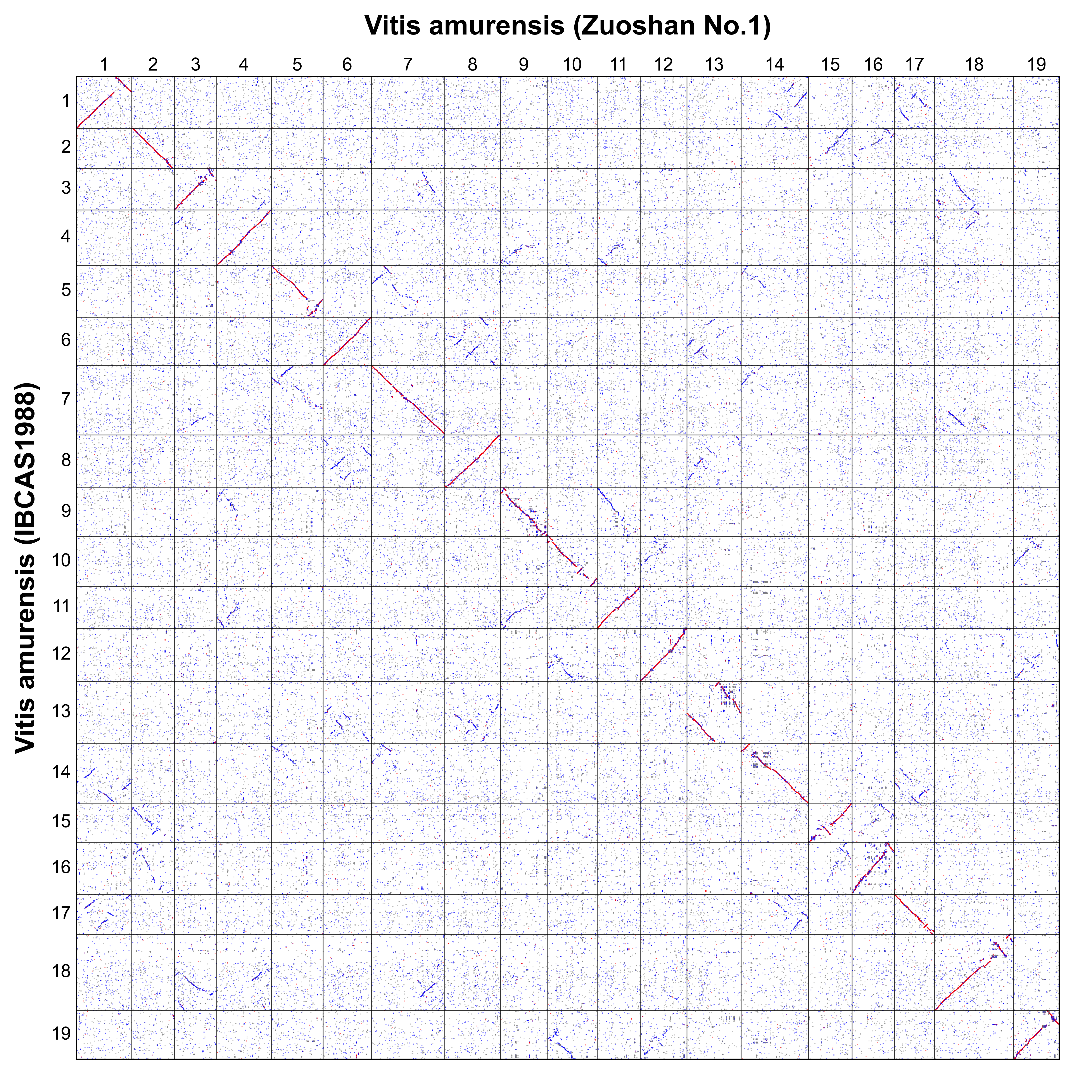
**

**Figure S1. Dotplot of homologous genes between *Va* Zuoshan No.1 and *Va* IBCAS1988 genomes**


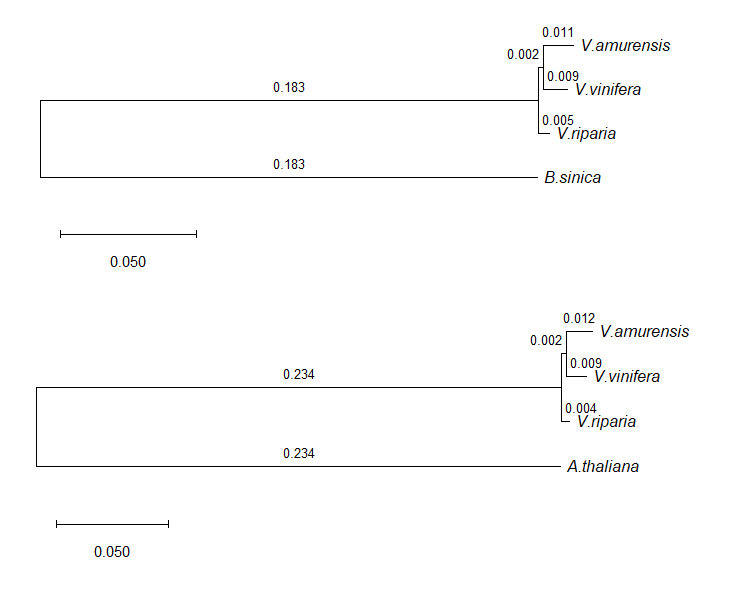


**Figure S2. The evolutionary tree based on the 3 grapes and Arabidopsis or Boxwood.**

**
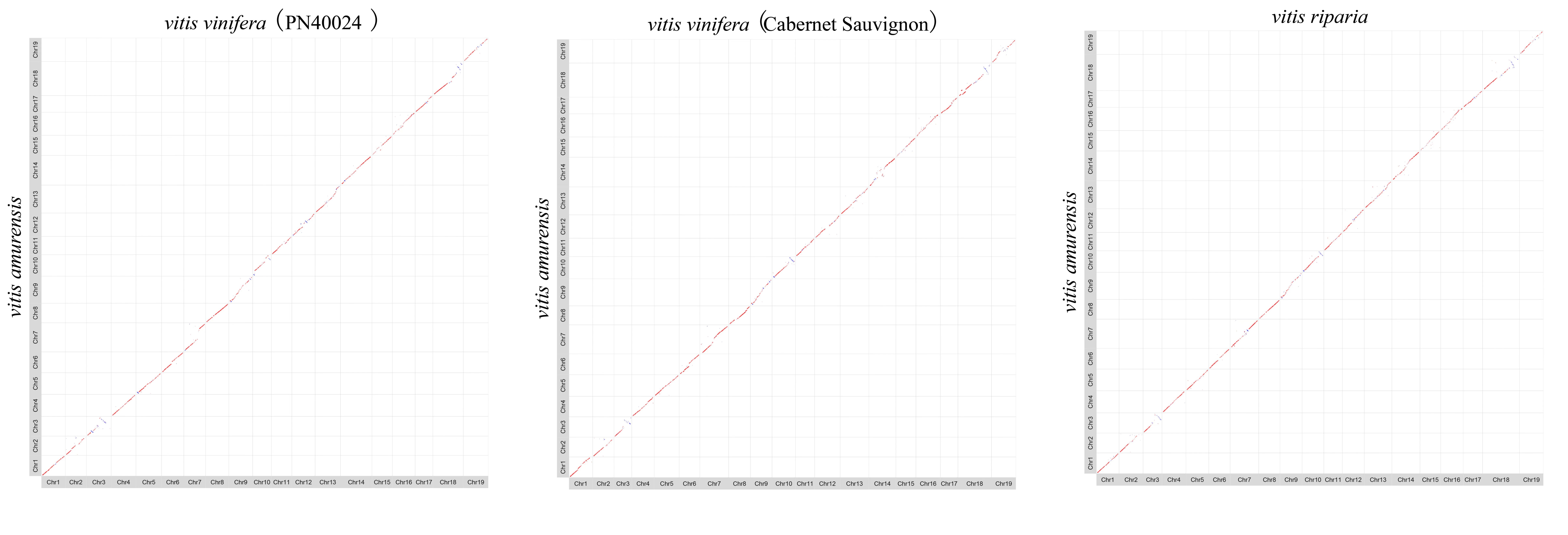
**

**Figure S3. Structural Variation analysis plot based on ggplot2.**


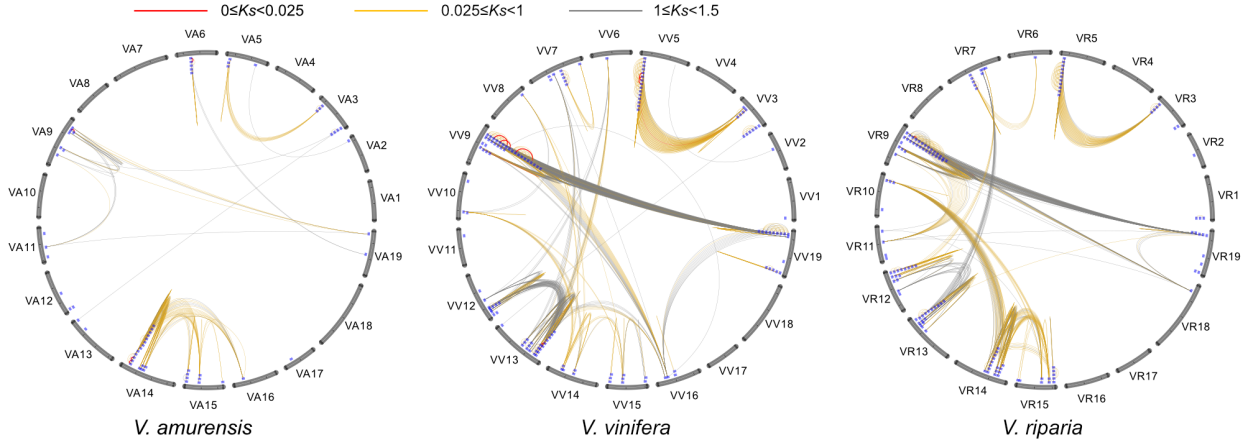


**Figure S4. The distribution of NBS family genes on the chromosomes of the three species. Lines represent Ks values of NBS gene pairs that are less than 0.025 (red), larger than 0.025 but less than 1 (orange), and larger than 1 but less than 1.5 (grey).**


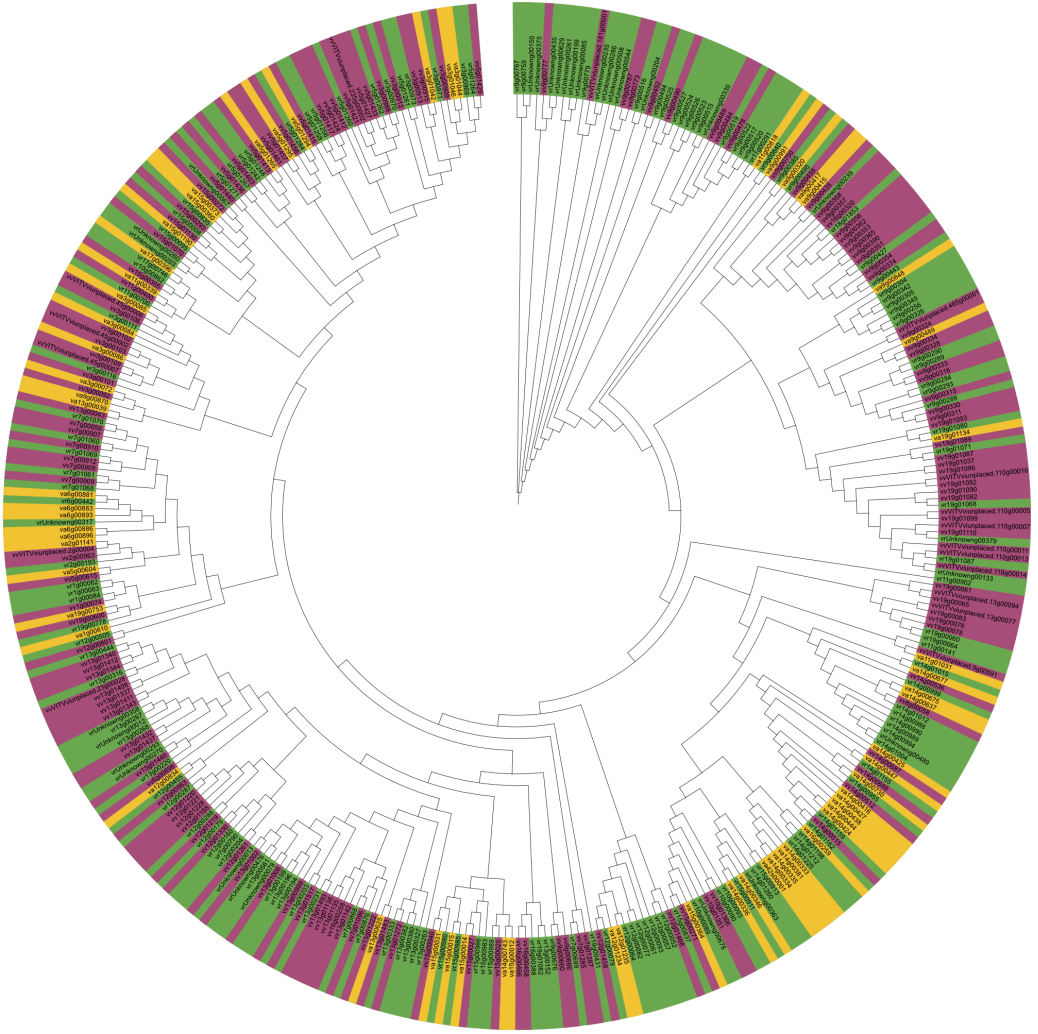


**Figure S5. Tree of NBS family genes of the three species.**

**
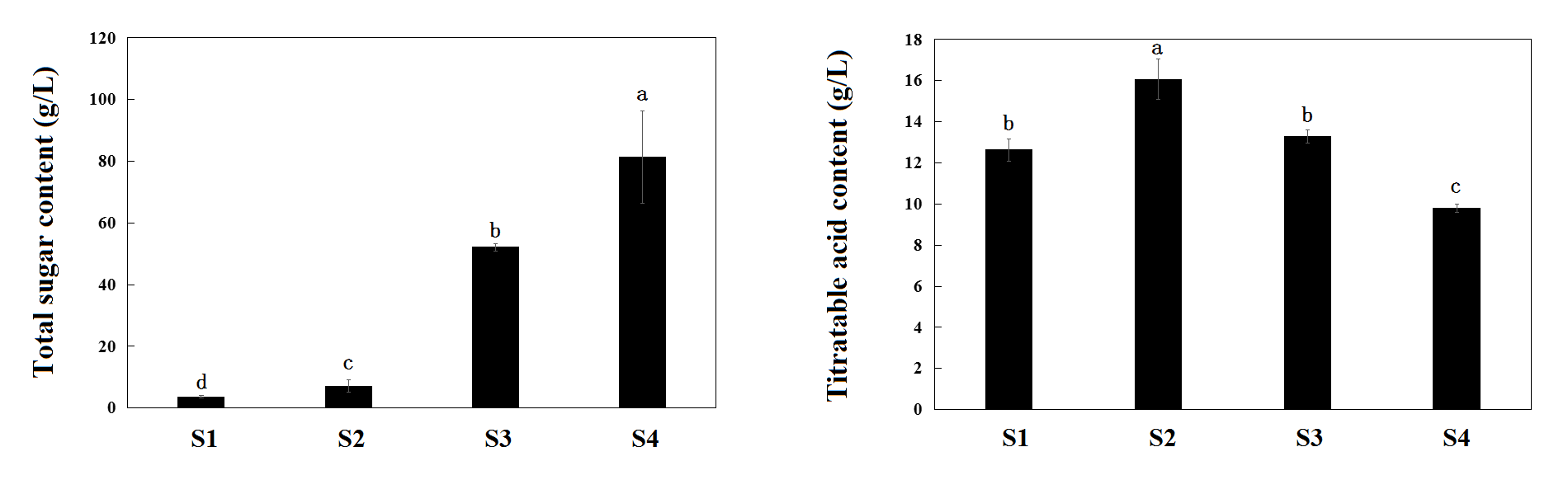
**

**Figure S6. The total sugar and titratable acid content of *Vitis amurensis* fruit at different stages.**


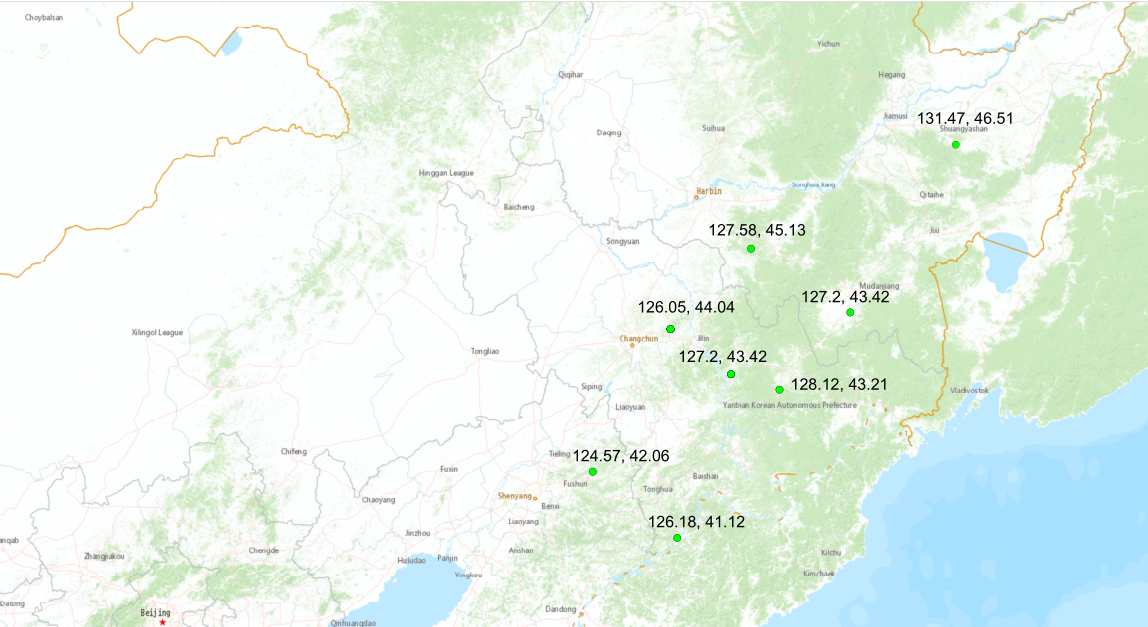


**Figure S7. Geological map for the 24 Va population. The green circle with latitude and longitude represents the origin of these 24 Va.**
